# Bioadhesive Controlled Metronidazole Release Matrix Based on Chitosan and Xanthan Gum

**DOI:** 10.3390/md8051716

**Published:** 2010-05-25

**Authors:** Ala’a F. Eftaiha, Nidal Qinna, Iyad S. Rashid, Mayyas M. Al Remawi, Munther R. Al Shami, Tawfiq A. Arafat, Adnan A. Badwan

**Affiliations:** 1 Suwagh Company for Drug Delivery Systems, A Subsidiary of the Jordanian Pharmaceutical Manufacturing Co., Naor, Jordan; E-Mails: alaachemistry@yahoo.com (A.F.E.); crl@jpm.com.jo (N.Q.); irashid@jpm.com.jo (I.S.R.); mayyas_nj@yahoo.com (M.M.A.R.); 2 Faculty of Pharmacy and Medical Sciences, Petra University, Amman, Jordan; 3 The Jordanian Pharmaceutical Manufacturing Company, Naor, Jordan; E-Mail: shabinda@hotmail.com (M.R.A.S.); 4 Jordan Center for Pharmaceutical Research, Amman, Jordan; E-Mail: aassad@e-dmn.com (T.A.A.)

**Keywords:** metronidazole, chitosan, xanthan gum, bioadhesion, bioavailability

## Abstract

Metronidazole, a common antibacterial drug, was incorporated into a hydrophilic polymer matrix composed of chitosan xanthan gum mixture. Hydrogel formation of this binary chitosan-xanthan gum combination was tested for its ability to control the release of metronidazole as a drug model. This preparation (MZ-CR) was characterized by *in vitro*, *ex vivo* bioadhesion and *in vivo* bioavailability study. For comparison purposes a commercial extended release formulation of metronidazole (CMZ) was used as a reference. The *in vitro* drug-release profiles of metronidazole preparation and CMZ were similar in 0.1 M HCl and phosphate buffer pH 6.8. Moreover, metronidazole preparation and CMZ showed a similar detachment force to sheep stomach mucosa, while the bioadhesion of the metronidazole preparation was higher three times than CMZ to sheep duodenum. The results of *in vivo* study indicated that the absorption of metronidazole from the preparation was faster than that of CMZ. Also, MZ-CR leads to higher metronidazole C_max_ and AUC relative to that of the CMZ. This increase in bioavailability might be explained by the bioadhesion of the preparation at the upper part of the small intestine that could result in an increase in the overall intestinal transit time. As a conclusion, formulating chitosan-xanthan gum mixture as a hydrophilic polymer matrix resulted in a superior pharmacokinetic parameters translated by better rate and extent of absorption of metronidazole.

## 1. Introduction

The simplest technique to prepare a controlled release matrix system is through direct compression of a single hydrophilic polymer with the drug substance. A wide variety of controlled release matrices have been developed. These include single component hydrophilic polymers such as hydroxypropyl methyl cellulose (HPMC), hydroxypropyl cellulose (HPC), sodium alginate, chitosan and xanthan gum [1–4]. However, depending on the system modifications, various drug release rates and patterns can be accomplished. The swelling mechanisms and kinetics of drug release from these systems are highly complex and therefore difficult to understand, as evidenced in previous publications [5–7].

It is evident that using single hydrophilic component has its shortcomings. Those for example, when polymer-retarding activity is only working at the outer layers of the tablet, with minimum hindering effect on drug release. Other shortcoming is the fluctuation of drug release at various time intervals [8]. Such drawbacks dictate the introduction of a second polymer modifying the drug dissolution from the first polymer. This concept was previously applied where a synergistic effect between xanthan and locust bean gums caused a more controllable drug release of a variety of drugs. This system is a mixture of natural hydrophilic polymer combination which gels in aqueous environment forming tight gel allowing precise predictable release patterns. Such system is commercialized under trademark TIMERx^®^ where nifedipine and oxybutynin are marketed in European markets as controlled release dosage forms [9–11].

Another interesting combination of hydrophilic polymers includes the use of a binary system composed of chitosan and xanthan gum. Chitosan is the linear cationic β(1–4) polysaccharide obtained by partial random deacetylation of chitin [4,12–14]. Xanthan gum is a heteropolysaccharide chain consists of two β-D-glucose units linked through the 1 and 4 positions. The side chain consists of two mannose and one glucuronic acid, so the chain consists of repeating modules of five sugar units. The side chain is linked to every other glucose of the backbone at the 3 position. About half of the terminal mannose units have a pyruvic acid group linked as a ketal to its 4 and 6 positions. The other mannose unit has an acetyl group at the 6 positions [1,15,16]. Xanthan gum, as a branched anionic polysaccharide, and chitosan form polyelectrolyte complexes that may function as a release-retarding barrier. The combination between xanthan gum and chitosan has been examined by different authors [17,18].

The residence time at different gastrointestinal sites represents a serious limitation that precludes the dosage form from optimization [19]. Hence, several attempts have been recently undertaken aiming at the prolongation of the gastric residence times. These include the use of floating drug delivery systems and polymeric bioadhesive systems, where some hydrophilic polymers has the property to adhere to biological membranes such as chitosan and xanthan gum [20–22]. Although the optimal controlled release ratio of binary mixture of chitosan and xanthan gum (*i.e.*, 1:1 w/w) was described previously [23–25] the bioadhesion of the binary mixture has not been systematically explored.

Consequently in this study, the assessment of bioadhesion of this controlled release matrix composed of binary mixture of chitosan-xanthan and its effect on metronidazole release *in vitro* and *in vivo* are described and compared with a commercially available metronidazole controlled release (CMZ) tablet based on a single retarding polymer (Leading Brand®).

## 2. Results and Discussion

Formulation of metronidazole controlled release tablets was based on the drug mixed with a 1:1 mass ratio between chitosan and xanthan gum. This ratio was based upon its suitability of use. At this ratio, the maximum interaction between chitosan and xanthan gum takes place, in addition, a good compressible (or flowable) and a highly compactable powder characteristics are attained at this ratio [25]. Therefore, industrial processing of this formulation is not expected to become a constraint when scaling-up is desired.

Hardness and friability were over 110 N and less than 0.5% for the uncoated MZ-CR tablets, respectively. Assay of metronidazole tablets was carried out in accordance to USP monograph. Metronidazole assay in the reference and test tablets were complying with USP monograph and found to be 101.65% and 100.34% for CMZ and MZ-CR, respectively.

### 2.1. In vitro release study

**Figure 1:**
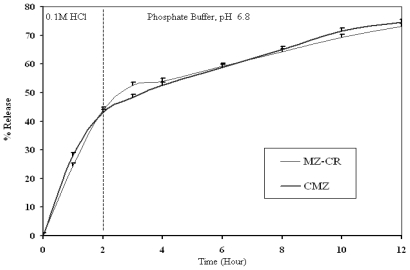
*In vitro* dissolution profile of MZ-CR and CMZ in a USP paddle apparatus using a dissolution medium of 900 mL of 0.1 M HCl for 2 hrs then USP phosphate Buffer, pH 6.8 for the rest period, 50 rpm and at 37 °C.

**Figure 2:**
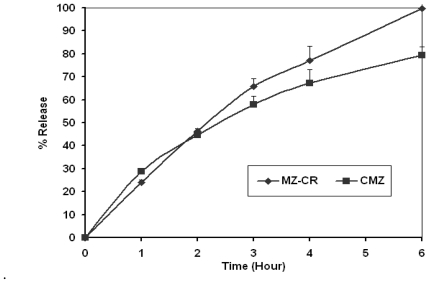
*In vitro* dissolution profile of MZ-CR and CMZ in USP paddle apparatus using a dissolution medium of 900 mL of 0.1 M HCl, 50 rpm and at 37 °C.

The *in vitro* dissolution profiles of MZ-CR and CMZ in 0.1 M HCl and in 0.1 M HCl/phosphate buffer, pH 6.8, are presented in Figures 1 and 2, respectively. A power law is used to describe drug release from polymeric system (1):

(1) $$Q={k\text{t}}^{n}$$

where *Q* is the percentage of drug release; *k* is a constant incorporating structural and geometric characteristics of the device, and *n* is the release exponent, which is indicative of the mechanism of drug release.

Table 1 represents *k*, *n* and the correlation coefficient of MZ-CR and CMZ in 0.1 M HCl of Figure 1 and in phosphate buffer pH 6.8 phase of Figure 2, according to the power law. As shown in Table 1, the drug release from the polymeric matrix in 0.1 M HCl seemed to follow anomalous transport in the case of CMZ and indicate a swelling-controlled drug release for MZ-CR. On the other hand, anomalous diffusion seems to be the release mechanism of MZ-CR and CMZ in phosphate buffer. Therefore, the mechanism of release, in the case of MZ-CR has changed from being swellingcontrolled to be anomalous diffusion. This could be explained by considering hydrogel formation. However, the rate constant (*k)* of MZ-CR was higher than that of CMZ in 0.1 M HCl. The release pattern of MZ-CR appeared to be almost similar to that of CMZ in 0.1 M HCl where the *f^2^* factor was 58.04%. Such similarity could be explained from the lower *n* value of CMZ than that of MZ-CR and the lower *k* value of CMZ than that of MZ-CR. While, in phosphate buffer the release kinetics were almost similar for MZ-CR and CMZ, where the *f^2^* factor for the *in vitro* data was 79.96%. Consequently, the two preparations have similar *in vitro* release behavior.

#### 2.1.1. Bioadhesion

**Figure 3:**
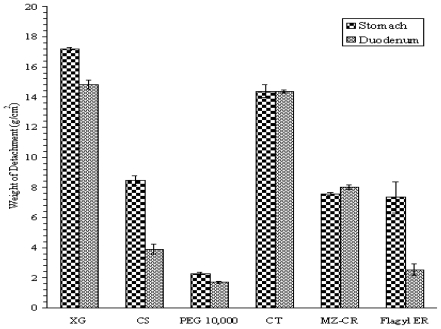
Weight of detachment per area (g/cm^2^) against each component in the formula of XG, CS, PEG, CT, MZ-CR, and CMZ.

The results of bioadhesion test performed on sheep stomach and duodenum for XG, CS, PEG, CT, MZ-CR and CMZ are presented in Figure 3. The bioadhesion of each component in the MZ-CR formulation was evaluated individually in order to elucidate the contribution of each component to bioadhesion. As shown in Figure 3, XG had the highest weight of detachment compared to that of CS and PEG 10,000. Moreover, the bioadhesion force of XG, CS, PEG and CMZ was higher in stomach than that in the duodenum, while the bioadhesion of CT and the MZ-CR formulation was almost the same in both the stomach and duodenum.

A comparison between MZ-CR and CMZ showed that both had a similar bioadhesion to stomach, while MZ-CR exerted a higher adhesion power to duodenum than CMZ. This could be explained by the formation of a hydrogel at media pH of the duodenum.

**Figure 4:**
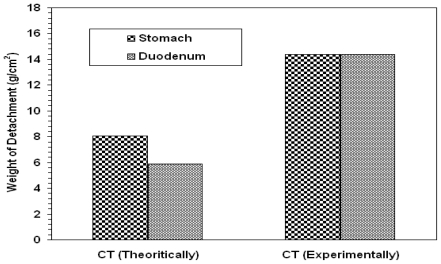
The calculated (theoretical) and the experimental values of weight of detachment per area (g/cm^2^) of a drug free tablet CT in stomach and duodenum.

However, for prediction purposes, the weight of detachment of CT was calculated theoretically according to additivity rule. The weights of detachment of CT theoretically and experimentally are shown in Figure 4. The results suggest that bioadhesion does not follow the simple additivity rule. Bioadhesion of the tablet with mucus tissue could be dependent on polymer concentration and/or availability of the surface. The polymer swellability is not the same for all polymeric components. Thus, their distribution and availability on the surface of the tablet might be modified once the tablet has been wetted. Accordingly, adhesion to the mucosa surface will be modified and the additivity rule will not be valid any more. This difference in bioadhesion might modify the gut transit time and may affect drug bioavailability as reported in such systems [26].

### 2.2. In vivo study

As shown in the summary table of the main pharmacokinetic metrics (Table 2), the absorption of metronidazole from the test product (MZ-CR) was faster than that of CMZ with a maximum plasma level attained at 4.37 and 6.14 hours, respectively. This relative faster absorption has resulted in significantly higher metronidazole maximum plasma for the test product (9.08 μg/mL) relative to that of the reference product (4.03 μg/mL). Trough levels at 24 hours post dose for both products after a single dose were almost identical and stand approximately at 1.8 μg/mL.

The faster absorption and the higher plasma concentration of metronidazole released from MZ-CR may be attributed to the adhesion characteristics of the hydrogel matrix causing a delay in its transit away from the upper part of the small intestine were absorption is anticipated to be optimum [27]. Furthermore, a significant difference in the area under the plasma concentration-time for MZ-CR and CMZ is illustrated. However, extrapolating the data to infinity resulted in a larger area under the curve for the CMZ compared to MZ-CR.

**Figure 5:**
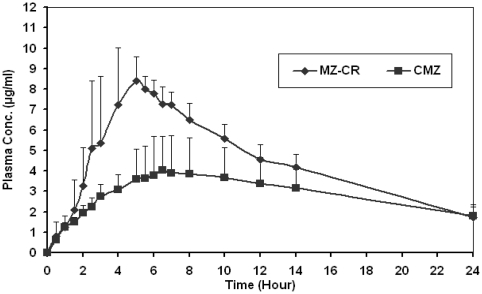
Mean plasma conc.time profiles following the administration of 750 mg metronidazole tablets for eight subjects (mean ± SEM).

This situation clearly suggests that comparisons based on values involving extrapolation must be undertaken with caution and would prove to be misleading and may produce unwarranted impact on the error term associated with the parameter being examined. Such impact is depicted by 90% exact confidence region where the area under the plasma concentration-time curve from time zero to infinity has been increased by more than five-fold in comparison to the area to the last sampling point. This situation could be rectified by the elimination of the most outlying subject. The coefficient of variation of CMZ was clearly much higher for all pharmacokinetic parameters in comparison with MZ-CR. This suggests that CMZ resulted in a higher variability in plasma concentration time profiles. Nevertheless, MZ-CR preparation gave a more reliable plasma concentration data as shown in Figure 5.

**Figure 6:**
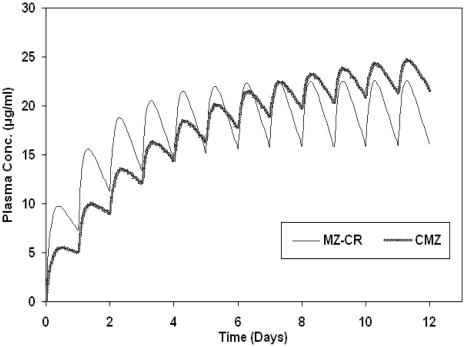
Accumulation of metronidazole in the plasma upon multiple dosing of 750 mg metronidazole every day for MZ-CR, CMZ and metronidazole immediate release (simulated).

The mean elimination half-life of MZ-CR and CMZ as estimated from the plasma concentration-time profiles are given at 22.7 and 49.9 hours, respectively. Since such findings have a significant effect on the accumulation characteristics of the formulation upon multiple dosing, a simulation study for such situation has been undertaken. The actual pharmacokinetic metrics estimated from the empirical data were used in the simulation shown in Figure 6. Multiple dosing usually results in accumulation of metronidazole until a steady state is attained. The time needed to reach a steady state was around four days for MZ-CR, while in case of CMZ it needs about 10 days to reach the steady state. Such impact could be better appreciated once the therapeutic index of metronidazole is defined.

Metronidazole is a drug that is used to treat infections caused by microbes. The clinical or pharmacological difference between MZ-CR and CMZ will be discussed in terms of metronidazole antimicrobial efficacy.

Metronidazole exhibits *in vitro* minimal inhibitory concentrations (MICs) of 8 μg/mL or less against most (≥90%) strains of the Gram-negative anaerobes such as the *Bacteroides fragilis* group (*B. caccae, B. uniformis*) and *Prevotella* species (*P. bivia, P. buccae, P. disiens*) microorganisms. However, the problem of metronidazole, as with most antimicrobials, is the appearance of drug-resistant strains. Many microbes have developed resistance to metronidazole, especially some strains of Helicobacter. Two *H. felis* strains showed a MIC of 16 μg/mL for metronidazole, suggesting acquired resistance to this antimicrobial agent [28,29].

The reason for the appearance of drug-resistant strains comes from incomplete eradication of bacterial infection by the antimicrobial therapy and/or the larger exposure time of bacterial pathogens to sub-inhibitory concentrations, which could lead to the selection of resistant strains of bacterial populations [30–32].

The antimicrobial efficacy is particularly dependent on the time that free plasma concentrations of the drug exceeding the minimum inhibitory concentration (MIC) for the target pathogen (T > MIC) [33]. It is well known that the increase in pathogen MICs (drug-resistant pathogens) can be overcome with increased unit doses, dose frequency and/or improved pharmacokinetics to maintain adequate T > MIC. Thus, in order to increase efficacy, there is a need to increase the total amount of drug given per dose [34].

This reveals the importance for the use of sustained release preparation that provides a constant level of antimicrobial drug above the MIC all over the therapeutic period. The achievement of high plasma level of antimicrobials may prevent the development of drug-resistant bacterial strains. This has been achieved by the increase in extent of drug absorption, and hence bioavailability, of MZ-CR in comparison with CMZ. The plasma concentration of metronidazole provided by MZ-CR was higher than CMZ all over their plasma concentration time profile for the entire period of 24 hrs, as shown in Figure 5. This means that microbes will be exposed to higher concentration of metronidazole for the entire period of 24 hr in MZ-CR preparation when compared to CMZ. This is important in order to maximize the antimicrobial agents efficacy against the development of resistant strains through the use of optimized kinetic profiles [35,36].

As conclusion, this study reveals the importance of carrying out *ex vivo* bioadhesion study for the evaluation of a newly developed controlled release matrix system in concurrent with *in vitro* release tests before conducting *in vivo* studies. Although the *in vitro* drug release studies indicated similarity in the release, the *in vivo* study showed a large difference in plasma concentration time profiles. On the other hand, the study indicates higher C_max_ of MZ-CR compared to CMZ due to the synergistic bioadhesion of the binary mixture of chitosan-xanthan gum on the upper part of the intestine.

## 3. Experimental

### 3.1. Materials

Pharmaceutical grade chitosan (CS) with a viscosity of 16.80 mPas (0.5% (w/v) in 0.1M HCl), having a 93% degree of deacetylation and average particle size of 170 μm, was purchased from Hong Ju, China. Food and pharmaceutical grade xanthan gum (XG) with a viscosity of 40.7 mPas (0.5% (w/v) in water) and average particle size of 106.7 μm, was purchased from Jungbanzlauer, Germany. Polyethylene Glycol (PEG) having molecular weight of 10 kDa was obtained from Sigma-Aldrich, Germany. Metronidazole (MZ) was purchased from Lution-Hong Gou, China. CMZ coated tablets were used as a commercially available metronidazole reference product purchased from the USA market. CMZ tablets contain 750 mg of metronidazole USP. Inactive ingredients include hypromellose, lactose, magnesium stearate, polyethylene glycol, poly(methyl)acrylic acid ester copolymers, polysorbate 80, silicon dioxide, simethicone emulsion, talc, titanium dioxide, FD&C Blue No. 2 Aluminum Lake.

### 3.2. Methods

#### 3.2.1. Preparation of MZ controlled release preparation (MZ-CR)

A powder mixture of 100 g containing 69.3 g metronidazole, 13.8 g polyethylene glycol and 8.45 g xanthan gum was granulated using 1% (w/w) solution of xanthan gum. The granulated mixture was passed on US mesh #4 sieve and dried in an oven at 45 ºC for 3 hours. Finally, the dry powder was sieved on a US mesh #22 sieve and mixed with 8.45 g of chitosan. The prepared granules were compressed into tablets containing 750 mg metronidazole using a single punch machine (Manesty, England). Punches with oblong shape (19 mm × 9.5 mm) were used.

#### 3.2.2. *In vitro* dissolution test

The *in vitro* release was performed using USP method II (paddle). The dissolution media in the first set of experiments was 0.1M HCl for six hours, while in the second set 0.1M HCl was replaced with phosphate buffer of pH 6.8 after 2 hours. The volume of dissolution media in each vessel was 900 mL and the temperature was maintained at 37 ± 0.5°C during the study. The paddle speed was adjusted to 50 rpm. Samples of 5mL were withdrawn at different time intervals (1, 2, 3, 4, 6, 8, 10, and 12 hours). Each withdrawn sample was replaced by 5 mL at each time of dissolution media. The percentage drug release was determined using of UV-spectrophotometer where λ_max_ was 276 nm in HCl and 322 nm in phosphate buffer respectively. A calibration curve was constructed and the concentration of metronidazole was read from that curve.

#### 3.2.3. Similarity factor

The dissolution profiles for MZ-CR and CMZ in different media were compared using Similarity factor (*f_2_*) suggested by FDA (1995) [37] and is described by Equation 2. Dissolution profiles of the reference and test products would be considered similar when *f_2_* is larger than 50:

(2) $$f_{2}=50\times \text{log}\;({\left[1+\text{Q}/\text{n}\right]}^{0.5}\times 100)$$

where Q equals the sum of squared differences between the percent released from the test and reference formulations at the sampling intervals (t = 1,...,n), where n refers to the number of sampling points.

#### 3.2.4. *In vitro* bioadhesion test

Bioadhesion was evaluated through the measure of maximum load necessary for the detachment of a tablet of defined composition from the surface of a sample of a biological tissue. The bioadhesion of tablets to sheep stomach and duodenum was measured using a modified two-arm balance method described by Parodi *et al.*, in 1996 [38]. The tablets were sandwiched between freshly prepared tissues of a sheep stomach or duodenum purchased from a local slaughterhouse. The tissues were cut into strips (2 × 3 cm) cleaned and wetted with normal saline. The upper tissue strip was placed under a movable plastic platform, while the lower strip was placed on a fixed stage.

Tablets were initially soaked in 0.1 M HCl for five minutes if stomach parts were used or in 0.1 M HCl and then placed in phosphate buffer (pH 6.8) for another five minute if duodenum parts were used. A preload of 10 g was placed above the sandwiched tablet for five minutes to establish adhesion. The preload was removed and water was pumped to a plastic container by a peristaltic pump at a rate of 10mL/min. The addition was stopped as soon as the detachment of the tablet from the mucosa. The weight of detachment was then calculated as the peak weight of the added water required for the detachment of the upper biological tissue from the tablet surface per area (calculated in g/cm^2^) was used as indicator for tablet bioadhesion.

This test was performed on drug-free compacts of XG, CS, PEG, CT (control tablets containing all additives), while MZ-CR and CMZ tablets were used as metronidazole containing compacts.

#### 3.2.5. *In vivo* assessment of MZ controlled release dosage form

A pilot study was undertaken to measure the rate and extent of absorption of 750 mg metronidazole in Test (MZ-CR controlled release preparation) and in CMZ. The *in vivo* study was approved by the Ethical Committee of the Ibn Al-Haytham Hospital in Amman, Jordan (where the study was conducted). Volunteers’ consents were obtained in concordance with the Declaration of Helsinki and Good Clinical Practice was followed throughout the study.

The study was conducted in accordance with an open, randomized, single-dose, two-treatments, two-periods, crossover design under fasting conditions. The hospitalization period was a total 24 hours including 12 hours before dosing, and 12 hours after dosing in each period. The dosing periods were separated by a washout period of sufficient length (7 days) which far exceeds ten elimination half-lives of the drug. The study consisted of eight healthy, 30–35 year old male participants. The metronidazole products were administered in the morning (at 8:00am) with 240 mL of water following a 12-hour fasting. A total of 20 blood samples were collected according the following sample collection schedule: 0 (20 mL pre-dosing) and 10 mL at 0.5, 1, 2, 2.5, 3, 3.5, 4, 4.5, 5, 5.5, 6, 7,8, 10, 12, and 24 hours after dosing.

All samples were collected in EDTA blood tubes, and centrifuged at 3,000 rpm for 4 minutes; plasma samples were transferred to screw top polypropylene tubes. These were capped and immediately stored at −30 ± 5 °C until analysis.

#### 3.2.6. Determination of metronidazole in plasma

A standard curve of metronidazole was prepared in plasma in the range of 0.10–15.0 μg/mL, sample preparation consisted of the addition of 0.2 mL of perchloric acid (10%) and 0.2 mL of 100 μg/mL of the internal standard (hydrochlorothiazide) and 50 μl of 4 M sodium acetate to 0.2 mL of plasma in eppendorf tubes, and vortexed for 1 minute. The supernatant was centrifuged for 10 minutes at 4,000 rpm and 50 μL sample was injected onto Hypersil phenyl column using 85% of potassium dihydrogen phosphate and 15% acetonitrile, the final pH was adjusted to 3.6. Metronidazole and the internal standard were separed from endogenous substances and the retention time was 3.0 to 4.0 minutes for hydrochlorothiazide at flow rate of 1.0 mL/minute. The lower limit of detection was 0.10 μg/mL.

#### 3.2.7. Pharmacokinetic analysis

The pharmacokinetic parameters were directly estimated from the plasma concentration-time data profiles. The elimination half-life (t_1/2_), expressed as ln(2)/Ke (Ke is the elimination rate constant), was estimated from the slope of the terminal segment of the plasma profile. The area from time zero to the last sampling point was also determined based on trapezoidal rule (AUC_0-tlast_).

#### 3.2.7.1. Evaluation of bioavailability

Relative bioavailability was determined based on the test to reference ratio (T/R) of geometric means of the main pharmacokinetic metrics of interest such as AUC_0-t_ and C_max_.

#### 3.2.7.2. Statistical considerations

The statistical treatment of data was based on a statistical model suited for a K-sequence and J-period crossover design:

(3) $${\text{Y}}_{\text{ijk}}=\mu +{\text{G}}_{\text{k}}+{\text{S}}_{\text{ik}}+{\text{P}}_{\text{j}}+{\text{F}}_{\left(\text{j}.\text{k}\right)}+{\text{C}}_{\left(\text{i}-1,\text{K}\right)}+{\text{e}}_{\text{ijk}}$$

where i = 1,...,nk; μ = the overall sample mean; J = 1,...,J; k= 1,...K; G, P and F are the respective fixed sequence (k), period (P) and formulation (F) effects; C_(i – 1, K)_ is the fixed first order carryover effect (C) of the treatment in the kth sequence administered in the (j – 1^th^) period; e_ijk_ is the within subject random error of Y_ijk_. The analysis of the variance for this model has been performed on the ln-transformed data. Consequently, a 95% two one-sided t-test (Schuirmann) as well as a 90% shortest confidence interval was established. Alternatively, average bioequivalence was assessed by constructing an exact (1 – 2α) × 100% confidence region.

## 4. Conclusions

The formulation of metronidazole with chitosan and xanthan gum hydrophilic polymers has proven to be a well representative example of a sustained release solid dosage form preparation. This was evident in the attained prolonged *in vitro* drug release, in the strong bioadhesivitiy attained when tested on a sheep duodenum, and in the high *in vivo* bioavailability obtained. When compared against a reference commercial metronidazole drug (CMZ), results indicated the superiority of such preparation over the CMS in all aspects of the *in vitro*-*in vivo* analysis.

## Figures and Tables

**Table 1:** Summary of power equation (Equation 1) components k, n and the correlation coefficient of MZ-CR and CMZ.

	MZ-CR	CMZ
	HCl	
K	29.48	25.37
N	0.8005	0.5789
r^2^	0.992	0.988
	Phosphate Buffer, pH = 6.8	
K	6.27	5.52
N	0.6654	0.7679
r^2^	0.997	0.997

**Table 2:** Summary of the main pharmacokinetic parameters for the test and reference products directly estimated from the plasma concentration-time data.

	T_max_		C_max_		T_0.5_		AUC_0-t_		AUC_0-∞_	

Vol. No.	T	R	T	R	T	R	T	R	T	R
1	5.00	4.00	7.96	2.62	18.74	151.10	91.74	43.81	123.65	372.97
2	5.50	10.00	7.08	3.16	20.64	44.15	81.53	52.71	122.63	119.59
3	3.00	6.50	11.93	4.75	16.73	23.84	107.45	70.89	132.07	115.95
4	4.00	5.00	9.28	3.25	27.42	50.73	121.67	63.73	214.62	213.04
5	5.00	6.00	8.98	8.24	23.91	27.09	108.18	112.73	179.23	212.39
6	4.00	5.00	9.32	3.93	22.61	31.77	97.77	63.60	147.04	125.93
7	5.00	8.00	9.11	3.27	27.89	93.25	103.72	57.19	185.00	332.97
8	4.00	6.50	9.75	5.19	26.45	59.22	124.67	71.48	213.97	276.53

G_Mean	4.37	6.14	9.08	4.03	22.71	49.88	103.70	64.67	160.86	201.46
STDEV	0.82	1.90	1.41	1.81	4.13	42.98	14.44	20.66	38.50	99.44
SEM	0.29	0.67	0.50	0.64	1.46	15.20	5.11	7.30	13.61	35.16
% CV	18.50	29.87	15.33	42.09	17.91	71.46	13.81	30.82	23.36	44.96
